# Are low birth weight neonates at risk for suboptimal renal growth and function during infancy?

**DOI:** 10.1186/s12882-016-0314-7

**Published:** 2016-07-26

**Authors:** A. Iyengar, S. Nesargi, A. George, N. Sinha, S. Selvam, V. A. Luyckx

**Affiliations:** 1 Department of Pediatric Nephrology, St John’s Medical College Hospital, Bangalore, India; 2 Department of Neonatology, St John’s Medical College Hospital, Bangalore, India; 3 Department of Radiology, St John’s Medical College Hospital, Bangalore, India; 4 St John’s Research Institute, St John’s Medical College Hospital, Bangalore, India; 5 Institute of Biomedical Ethics, University of Zurich, Zurich, Switzerland

**Keywords:** Low birth weight, Small for gestational age, Renal volume, Cystatin C-derived glomerular filtration rate

## Abstract

**Background:**

To assess the renal growth and function of neonates during infancy in relation to birth weight and gestational age.

**Methods:**

A longitudinal study was conducted at a tertiary hospital in South India from June 2010 to August 2014. Low birth weight neonates (LBW) were further sub-classified based on gestational age and compared with normal birth weight (NBW) full term neonates at birth, 6 months and 18-24months of age. The renal volume was measured by ultrasound and renal function by Cystatin C- derived glomerular filtration rate (CysGFR) at the three time points during the dynamic phase of renal maturation in infancy.

**Results:**

We recruited 100 LBW and 66 NBW term neonates. Thirty five percent of the LBW neonates were SGA. Among the AGA neonates, 39 % were LBW neonates. The mean height and weight of the LBW neonates were significantly lower compared to NBW neonates throughout infancy. The increment in kidney volume was in accordance with the change in body size, being lower in LBW compared to NBW infants. The combined kidney volume was significantly lower in LBW and SGA neonates across all three time points (*p* < 0.001). CysGFR in the LBW and SGA infants, despite having low kidney volumes, were comparable to the GFRs of NBW and AGA neonates at the end of infancy.

**Conclusion:**

This study highlights the fact that both birth weight and gestational age influence kidney growth and function in infancy. At the end of infancy, despite a significant difference in kidney volumes and age at last follow up, the glomerular filtration rate was comparable between LBW and NBW infants. Though not statistically significant, there was a trend towards higher urine microalbumin in LBW compared to NBW in infancy.

**Electronic supplementary material:**

The online version of this article (doi:10.1186/s12882-016-0314-7) contains supplementary material, which is available to authorized users.

## Background

India is the cradle for 40 % of all low birth weight babies in the developing world [1]. LBW has been identified as an independent risk factor for progression of renal diseases, hypertension and diabetes mellitus in adult life [2–5]. Studies on neonates suggest that Indian babies are born smaller but are relatively fatter compared to Caucasian babies and are referred to as the” thin fat Indian baby” [6]. This thin fat phenotype persists in childhood and is a forerunner of diabetes and metabolic problems in adulthood [6, 7]. From the developing world, landmark studies have affirmed the associations between birth size and later risk of disease [8, 9]. The increase in burden of cardiovascular disease in India, especially affecting people between ages 25-69years is alarming [10]. With a population of >1 billion, the age adjusted incidence rate of ESRD in India is 232 per million population with >275,000 patients reach ESRD annually, with a majority remaining un- dialysed [11].

LBW babies have a lower nephron mass [12]. Fetal kidney development begins at nine weeks and is completed by thirty six weeks. No new nephrons are formed after birth at term gestation. Risk factors for low nephron mass and /or kidney dysfunction are low birth weight, prematurity, low kidney mass and volume and gene polymorphisms [13]. The clinical correlation of low nephron mass with increased risk of hypertension and renal disease is known. Renal mass and glomerular number correlate significantly in normal adults and infants [13]. Renal volume is proportional to renal mass and therefore this has been used as an in vivo surrogate for the estimation of renal mass and therefore of glomerular number [14]. This renal volume may be measured by ultrasound in children including in neonates [15]. A small kidney on ultrasound may imply low nephron mass but an increase in kidney size cannot distinguish between normal growth and hypertrophy.

LBW could be secondary to intrauterine growth restriction (SGA) or due to prematurity(AGA). It is well established that weight for gestational age is an important independent determinant of kidney size at birth and early infancy [16]. There is data on LBW neonates, achieving similar glomerular filtration rates(GFR) compared to normal weight neonates despite 25 % smaller kidney volumes in the first week of life [17]. South Asian babies seem to have smaller kidneys compared to British babies even after adjusting to confounding factors influencing birth weight [18]. However, there is scanty information on the relationship between kidney function and kidney volume in LBW infants from birth to late infancy, which is the crucial and dynamic phase of renal functional maturity. There is a need to focus on the critical issue of nephron protection at birth, emphasising the importance of regular monitoring of GFR in high risk infants based on critical factors such as birth weight and gestational age [19].

Though nephron number is a non-modifiable factor, for the clinician increased awareness may lead to changes in practice that could have far reaching consequences. Therefore, evaluating the renal function and growth in LBW infants becomes vital during the dynamic phase of renal growth and maturity of infancy.

The objective of this study was to primarily assess renal growth and function in LBW neonates compared to NBW neonates during infancy and secondarily to compare the renal growth and function in SGA and AGA newborns based on gestational age.

## Methods

This longitudinal study was conducted at St. Johns Medical college hospital, Bangalore from June 2010 to August 2014. Approval from the Institutional Ethical Review Board was obtained. Informed consent was taken from either of the parents of the newborn babies. NBW babies born at full term served as controls. LBW babies, born both intramural and extramural were included. The LBW babies consisted of term SGA, preterm SGA and preterm AGA. LBW was defined as a birth weight of < 2500 g. Prematurity was defined as any baby born before 37 completed weeks of gestation. Gestational age [20] was determined by a first trimester ultrasound or by New Ballard Score (done before 7 days of life). Babies were classified as SGA and AGA based on Lubchencos charts.

Babies with congenital anomalies of kidneys, syndromic associations, birth weight <1000gms (extremely low birth neonates), serum creatinine >1 mg/dl, hypertension, single kidney and abdominal pathology that made ultrasonography difficult to perform were excluded from recruitment. Weight was checked on a digital weighing machine to the nearest 5 g, length was measured on a kidimeter to the nearest 0.1 cm. Weight, length renal function and renal volumes were measured at birth, 6 months and between 18–24 months.

### Kidney volume assessment [21]

Kidney size was determined by ultrasonography (digital Sonoline G20 system) using a 5 MHz sector probe by an experienced ultrasonographer. The kidneys were identified in the sagital plane along the longitudinal axis. An average of the maximal length, transverse and anterior-posterior diameter was measured at the level of renal hilum to the nearest 0.1cms in both kidneys. Each measurement was made three times and averaged to minimize measurement error. The inter-observer and intra-observer interclass coefficients were 0.98(0.91-0.99) and 0.99(0.98-0.99) respectively. The renal sinus was eliminated to record accurately the dimensions of the renal parenchyma. Renal volume was calculated using an equation of an ellipsoid: Length x transverse diameter x anterior-posterior diameter x 0.523 (cm3). The combined renal volumes was obtained by adding the left and right renal volumes. The relative kidney volume was calculated as combined renal volume corrected to BSA to eliminate the birth weight as a confounding factor.

### Glomerular filtration rate (GFR) calculation [22, 23]

Serum cystatin C is a better measure of GFR than serum creatinine in infants, since serum creatinine is influenced by infant’s muscle mass and hydration status. Cystatin C was measured using the N Latex cystatin kit (Dade Behring) by nephelometric assay with reference range of 0.53 to 0.95 mg/l.GFR was estimated from cystatin C levels (CysGFR) using the prediction equation (log cystatin GFR = 1.962+ (1.123 X Log (1/cystatin C).

### Urine microalbumin

The microalbumin was measured using a Flex reagent cartridge is based on a particle –enhanced turbidometric inhibition immunoassay (PETINIA) adapted to Dimension clinical chemistry system which allows direct quantification of albumin in urine samples. The analytical measurement range is 1.3 -100 mg/l.

### Statistical methods

Descriptive statistics were reported using mean and standard deviation. Assumption of normality was checked. Figures are presented as box plots and scatter plots. The independent *t*-test was used to compare the continuous variables such as body weight, body surface area, serum cystatin C, CysGFR and combined renal volumes between LBW and NBW neonates at birth. Pearson or Spearman’s Rho correlation was used to assess the relationship between clinical variables for each time point. In order to predict the expected growth of kidney volume and function in LBW and NBW babies, multiple regression analysis was performed and regression coefficients (the average change in the dependent variable for every unit change in the independent variable) are reported. Mixed model analysis was performed to compare the mean kidney volume and function overtime separately between LBW and NBW neonates, considering study group (LBW/NBW and SGA/AGA) and time (at birth, 6 and 18 months) as factors adjusting for sex, BSA (for kidney volume) and kidney volume (for CysGFR). An alpha level of less than 0.05 was set as statistical significance. All analyses were carried out using SPSS version 21(IBM SPSS Statistics for Windows, Version 22.0. Armonk, NY: IBM Corp).

## Results

We screened 142 low birth weight babies. Of these, 100 met inclusion criteria and agreed to come for follow up (42 had developed illness needing neonatal intensive care). The controls consisted of 66 normal weight term neonates. All these babies recruited at birth were followed at 6 months and 18–24 months. Of the 166 neonates, 53 % were males. The mean age of infants at last follow up varied between the LBW and NBW neonates (LBW: 17.84 ± 4.6Vs NBW: 23.5 ± 2.7 months *p* < 0.01) but was not significantly different between SGA and AGA neonates (SGA: 17.3 ± 5.0 Vs AGA: 19.5 ± 5.1 months *p* > 0.05). Gender distribution was comparable between LBW and NBW groups.

Overall, the mean age of the mothers and the gestational age of the babies were 24 ± 4 years, and 36 ± 3 weeks respectively. The mean age of the mothers were comparable between LBW and NBW groups (LBW: 24.2 ± 3.5yrsvs. NBW: 23.7 ± 3.6 yrs). Thirty five percent of the LBW neonates were small for gestational age. Among the AGA neonates, 39 % were LBW.

**Fig. 1:**
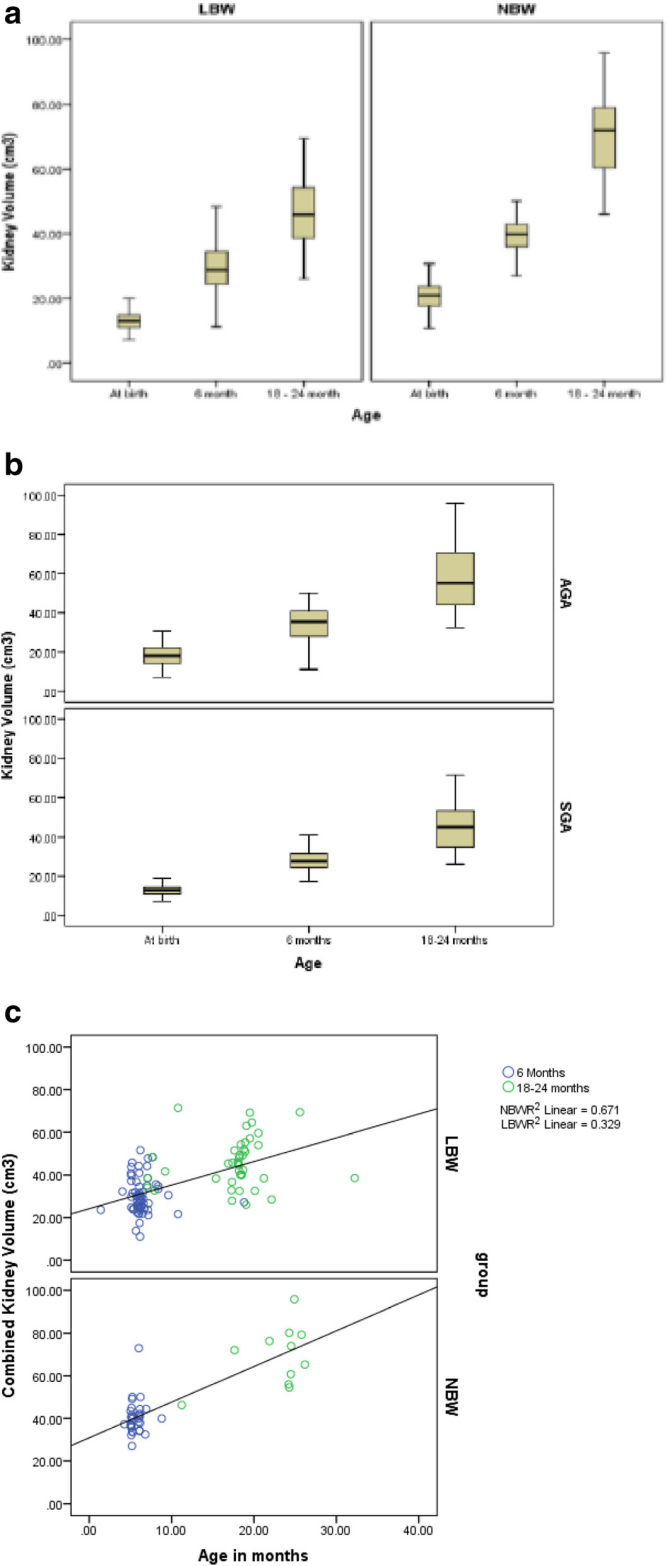
**a**: Kidney volume at 0,6 and 18–24 months in LBW and NBW neonates at all 3 time points. **b**: Kidney volume at 0,6 and 18–24 months in SGA and AGA neonates at all 3 time points. **c**: Rate of growth of kidney volumes over 18-24months in LBW and NBW neonates

The mean height and weight of the LBW neonates were significantly lower compared to NBW neonates over time. The profile of body weight, body surface area, serum cystatin C, CysGFR, combined renal volumes and urine microalbumin are depicted in Tables 1 and 2 over three time points in both NBW and LBW neonates and SGA versus AGA neonates respectively. The combined kidney volume was significantly lower in LBW at all three time points (*p* < 0.001) (Fig. 1a). The kidney volumes were also higher among AGA compared with SGA babies at all three time points (*p* < 0.001) as shown in (Fig. 1b). NBW neonates had a higher kidney volume compared to LBW neonates even after correcting for the body surface area (*p* < 0.001). There was a significant increase in kidney volumes in both groups over time (Fig. 1c as shown in the Additional file 1). The increment of renal growth was higher in NBW neonates (β coefficient of 0.57) compared to LBW neonates (β coefficient of 0.31). Similarly, increment in the renal growth was higher in AGA babies (β coefficient 0.95) and SGA babies (β coefficient 0.57). The relative growth rates of kidneys in LBW and SGA babies over time were slower than their counterparts.

**Table 1:** Comparison of anthropometry, kidney growth and function between LBW and NBW neonates

Parameters	LBW (*n* =100)	NBW /Controls (*n* = 66)
Weight (kg)		
Birth	1.65 ± 0.38	2.96* ± 0.38
6 months	5.76 ± 1.19(n = 66)	7.53* ± 1.54(n = 28)
18-24 months	9.31 ± 1.11(n = 46)	10.7* ± 1.56(n = 19)
BSA (m2)		
Birth	0.14 ± 0.02 (100)	0.20* ± 0.02 (64)
6 months	0.31 ± 0.07 (66)	0.38 * ± 0.06 (28)
18-24 months	0.41 ± 0.10 (46)	0.48 * ± 0.08 (17)
Combined renal volume (cm3)		
Birth	13.29 ± 3.38 (99)	20.58* ± 4.59 (65)
6 months	30.16 ± 8.19 (66)	40.27* ± 7.97 (32)
18-24 months	45.83 ± 13.26 (45)	69.61* ± 13.12 (19)
Cystatin GFR (ml/m^2^/min)		
Birth	51.63 ± 14.14 (95)	48.48 ± 8.29 (36)
6 months	64.98 ± 14.47 (66)	75.34* ± 12.56 (31)
18-24 months	75.80 ± 18.31 (45)	86.38 ± 15.61 (16)
Urine microalbumin†(mg/l)		
6 months	11.3 (4.9, 16.3)	11.2 (5.8, 14.0)
18-24 months	11.8 (5.8, 14.0)	10.1 (5.6, 12.5)

Reported as mean ± SD; † reported as median (25^th^ and 75^th^ percentiles)

*p <0.05, using an independent *t* test between LBW and NBW neonates

**Table 2:** Comparison of kidney growth and function between SGA and AGA neonates

	SGA	AGA
Combined renal volume (cm^3^)		
Birth	13.25 ± 3.54 (n = 58)	18.14 ± 5.31* (n = 106)
6 months	30.11 ± 7.92 (37)	35.19 ± 9.93* (61)
18- 24 months	45.58 ± 13.16 (24)	57.83 ± 16.07* (40)
Cystatin GFR (ml/m^2^/min)		
Birth	50.21 ± 6.73 (55)	78.39 ± 30.27* (75)
6 months	62.96 ± 12.63 (38)	71.67 ± 14.97 *(58)
18- 24 months	77.58 ± 16.24 (21)	79.47 ± 19.39 (*33)
Micro albuminuria (mg/L)†		
6 months	12.84 ± 10.35 (37)	12.73 ± 10.80 (55)
18- 24 months	12.56 ± 6.03 (21)	9.29 ± 5.24 (36)

Reported as mean ± SD; *p < 0.05, using an independent *t* test between SGA and AGA groups

† − comparison of microalbuminuria was done using Mann–Whitney *U* test

**Fig. 2:**
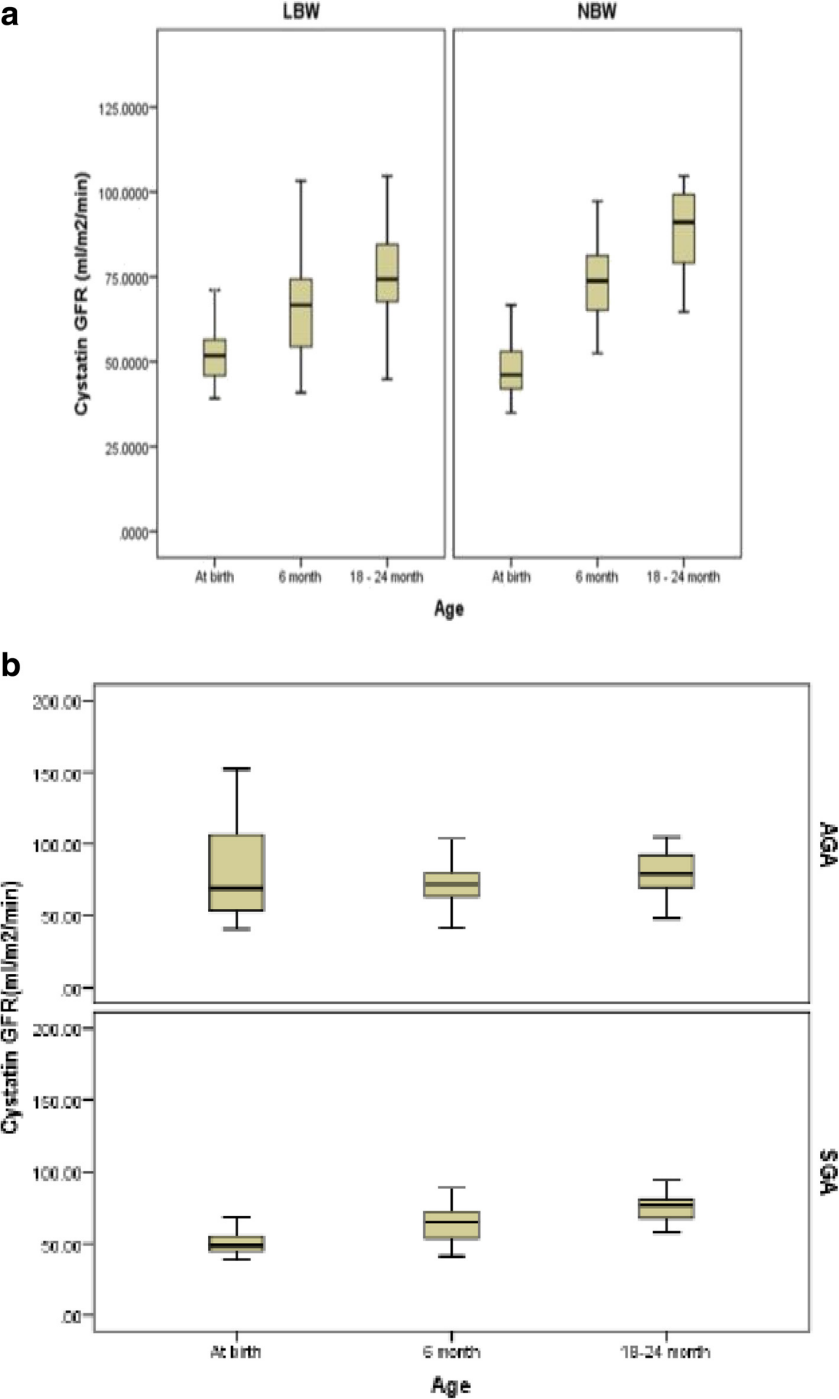
**a**: Cystatin C derived GFR between LBW and NBW neonates at all 3 time points. **b**: Cystatin C derived GFR between SGA and AGA neonates at all 3 time points

**Fig. 3:**
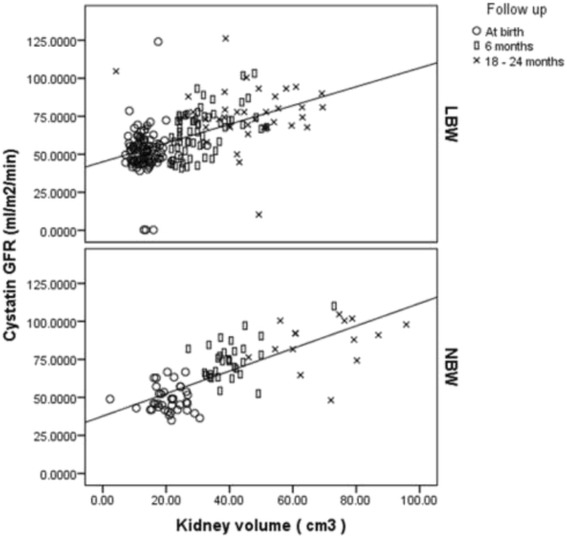
Correlation of kidney volume and kidney function in LBW and NBW during infancy

The CysGFR (ml/min/m^2^) at birth were 51.4 ± 14 and 48.5 ± 8.4 in LBW and NBW neonates respectively. CysGFR in LBW neonates were comparable to NBW neonates at the end of infancy (Fig. 2a) despite having lower kidney volumes. Similarly CysGFR was comparable between AGA and SGA at the end of infancy (Fig. 2b). Correlating kidney volumes and kidney function (Fig. 3), overall, there was a significant increment in the CysGFR in both LBW and NBW overtime, and however there was no significant group and interaction effect observed after adjusting for kidney volume.

There was no significant gender difference in any of the parameters between groups. Although the mean microalbuminuria at 18 months among LBW (11.0 ± 5.8 mg/l) and SGA (12. ± 6 mg/l) babies were higher than NBW (9.3 ± 5.3 mg/l) and AGA (9.2 ± 5.2 mg/l) babies, this was statistically not significant (Table 1 and 2). No infant in either group had pathological albuminuria above 30 mg/l.

## Discussion

Over the last 20 years, numerous studies have confirmed the association between low nephron number and renal disease [2, 12, 24]. After birth, GFR increases rapidly relative to kidney weight, body size and surface area until the GFR, corrected for body surface area reaches adult values by 2 years [25]. We therefore studied kidney growth and change in GFR during this dynamic period in a cohort of South Indian infants. Kidneys in LBW and SGA infants remained small and grew more slowly when compared to kidneys of NBW or AGA infants during the first two years of life. GFRs were however comparable by 18–24 months, suggesting relative hyperfiltration in the smaller kidneys which may be a precursor of adult disease.

### Kidney growth

Few studies have tracked kidney growth from early fatal life or birth to late infancy. The Generation R study prospectively studied kidney growth from fetal life until late infancy, and found that fetal biometrics and maternal anthropometrics were associated with kidney size at 2 years of age [26]. A large prospective study of NBW healthy infants, from fetal life to 2 years post-natal age found that kidney size was influenced by age and gender [27]. Kidney growth from 0–18 months of age was studied in 178 children born pre- or post-mature and/or small or large for gestational age, comparing them to 717 term AGA children. Relative kidney growth was positively correlated with weight for gestational age in this study [14]. Twenty years after birth,51 individuals born preterm, either SGA or AGA were compared with 30 full term controls. Both absolute and relative left kidney length and volume were significantly lower in SGA and AGA individuals, more notably in women [28]. Consistent with the literature therefore, tracking the kidney growth from birth to late infancy, our observations reveal that kidney size and volume are influenced by both birth weight and gestational age in Indian cohort infants from birth to 2 years. Importantly, the rate of change in kidney volume (β co-efficient) was lower in infants born with LBW and SGA, suggesting relatively less catch-up in renal size compared with body growth.

### Kidney function

In a retrospective study of normal children, a strong correlation was reported between renal function based on creatinine and renal mass [29]. However, in early childhood, when GFR was estimated using creatinine, a clear and consistent difference between AGA(n27) and SGA(n 39) infants was not found [30]. Vanpee et al. found that GFR was not different between LBW and NBW children at the age of 8 years, whereas, other studies have observed a lower GFR and poorer tubular function in LBW children aged6 -12 yrs [31–33]. In LBW infants and malnourished children, cystatin C was found to reflect GFR better than creatinine [34, 35]. We used the cysGFR to evaluate renal function in our cohort to avoid limitations with serum creatinine in this high risk group of neonates. Our results show that renal function at age 18–24 months was independent of renal size, birth weight or gestational age. Although kidneys were smaller in LBW and SGA infants, GFRs were not consistently different, suggesting that renal size is not the only determining factor for renal function. The variability in GFR measurements between studies may also reflect differences in the source population studied, i.e. ethnic, geographic, socioeconomic and dietary components, or may reflect hyperfiltration in a smaller kidney.

### Renal growth and Renal function

In a study [36] undertaken by us in 2006,comparing a cohort of babies from Canada (n =47) and India(*n* = 46),despite no significant difference in birth weights, the mean combined renal volume, corrected to body surface area, of Indian babies at 1 month of age was significantly (*p* < 0.001) smaller than that of Canadian babies. Focusing on the relationship between glomerular filtration rate and kidney volume in LBW neonates in early post- natal period, Kandaswamy et al. reported that within 6 days of birth, LBW infants, though small in number (*n* = 13) achieved a similar GFR to NBW infants (*n* = 36), despite 25 % smaller kidney volumes [17]. We also found no difference in GFRs between LBW and NBW groups at birth. In our cohort, although GFRs were significantly lower among LBW infants at 6 months, this difference was no longer evident at 18–24 months, suggesting possible development of compensatory hyperfiltration over time as the renal size relative to body size remained lower in the LBW group. Studies have shown evidence of hyperfiltration based on presence of urine microalbumin in early adulthood rather than in childhood [13, 37]. This surrogate marker of single nephron GFR or hyperfiltration in the form of microalbuminuria, though not statistically significant in our study, showed a higher trend in LBW/SGA compared to NBW/AGA babies at the end of infancy.

We analyzed the rate of growth of renal volume per time for NBW and LBW. The rate of growth was greater in NBW neonates at both 6 and 18 months as compared to LBW neonates. Mixed model analysis was performed and we found that the rate of growth was significantly different between NBW and LBW neonates (β coefficient for LBW = −10.4; 95 %: −15.8 to −4.9). However, we are cautious in interpreting these findings due to the drop outs that resulted in small sample size and the different ages at final kidney size measurement.

Our study has several limitations. Over time, drop out of patients resulted in smaller numbers in the later age groups, which may have confounded our results. Because of the smaller numbers at later time points we could not further stratify by gestational age within the two birth weight groups. The ages at study were somewhat variable with birth parameters being measured between birth and 14 days and the latest parameters measured between 18 and 24 months of age. We observed that the parents of LBW/SGA infants were sensitized to report on time or earlier for follow up compared to NBW babies in late infancy. It is possible that the lower age at last measurement in the LBW group may have contributed to the smaller kidney size in this group, however our regression analysis over time show a consistently reduced rate of renal growth in the LBW group which would support smaller final kidney sizes. Importantly also, despite there being a difference in the mean ages at last follow up between 18–24 months in LBW and NBW babies, the CysGFR was not different between the two groups at this time point. We did not have data on other factors that may have influenced infant renal growth and function such as maternal factors, nutrition and genetic predisposition, and therefore other potential confounders could not be controlled for.

Strengths of our study include the relatively large subject numbers, as well as the fact that this study was embedded within our day to day practice and therefore is likely generalizable to our population. In addition the repeated measurements over time and consistencies of trends underscore the validity of our findings as compared to single cross-sectional measurements. Our use of CysGFR is an important strength in that it reduces potential confounders introduced by serum creatinine and is one of only a few studies in neonates that use cysGFR estimation. The variability of findings in the literature especially with respect to GFR in LBW compared with NBW children demonstrates the challenges in studying GFR in these infants, where true GFR measurements as mentioned in literature [38] may not be considered ethical purely for study purposes. Our findings therefore add to the richness of this data, but more studies are required in Indian population to assess potential additional factors impacting GFR in infancy.

## Conclusion

Our study focuses on a high risk group of neonates with LBW and SGA, which is of great relevance in developing nations. Our assessment of renal growth and function during the dynamic phase of body growth and glomerular functional maturation in infancy, demonstrates that renal growth, in accordance with body growth, is suboptimal in LBW and SGA neonates compared to NBW and AGA neonates during infancy. Renal function however was comparable between LBW and NBW neonates and similarly between SGA and AGA neonates in late infancy. This catch up in glomerular filtration rate despite having lower kidney volumes may reflect early glomerular hyperfiltration and therefore emphasises the need for long term follow up of LBW and SGA infants.

## Abbreviations

AGA, appropriate for gestational age (babies’ whose birth weight is between the 10^th^ and 90^th^ centile for that gestational age); cycGFR, Cystatin C derived glomerular filtration rate; GFR, glomerular filtration rate; LBW, low birth weight (birth weight < 2500 g); NBW, normal birth weight; SGA, small for gestational age (babies’ whose birth weight is < 10^th^ centile for that gestational age)

## Additional file

Additional file 1: Graph showing rate of change in kidney volumes over time in the two groups- low birth weight and normal birth weight. (DOCX 42 kb)
