# Supplementary material for: Are low birth weight neonates at risk for suboptimal renal growth and function during infancy?
Source: BMC Nephrol. 2016 Jul 26;17:100. doi: 10.1186/s12882-016-0314-7 (PMC4962347; doi:10.1186/s12882-016-0314-7)

**Supplementary files:**

Graph showing rate of change in kidney volumes over time in the two groups- low birth weight and normal birth weight .

We analyzed the rate of growth of renal volume per time for NBW and

LBW. As depicted in the graph below, the rate of growth was greater in NBW neonates

at both 6 and 18 months as compared to LBW neonates. Mixed model analysis was

performed and we found that the rate of growth was significantly different between NBW and LBW neonates (coefficient for LBW = -10.4 ;95%: -15.8 to -4.9).

Supplementary data Figure 1c: Rate of growth of kidney volume over time in LBW and NBW


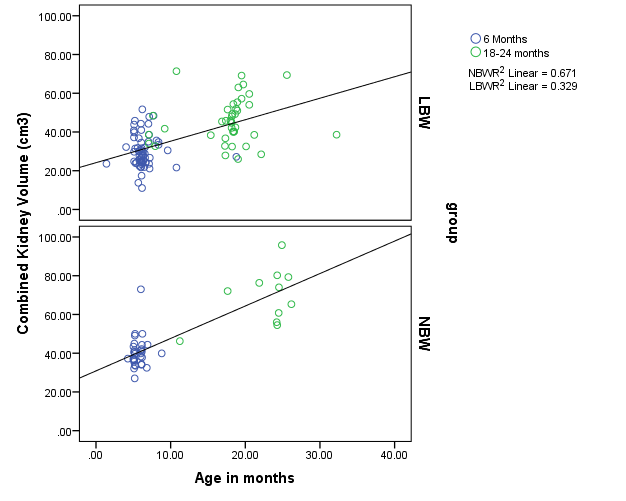

Supplement: Additional file 1: — Graph showing rate of change in kidney volumes over time in the two groups- low birth weight and normal birth weight. (DOCX 42 kb) [file 12882_2016_314_MOESM1_ESM.docx]
